# Using Data-Driven Learning to Predict and Control the Outcomes of Inorganic Materials Synthesis

**DOI:** 10.1021/acs.inorgchem.3c02697

**Published:** 2023-09-28

**Authors:** Emily M. Williamson, Richard L. Brutchey

**Affiliations:** Department of Chemistry, University of Southern California, Los Angeles, California 90089, United States

## Abstract

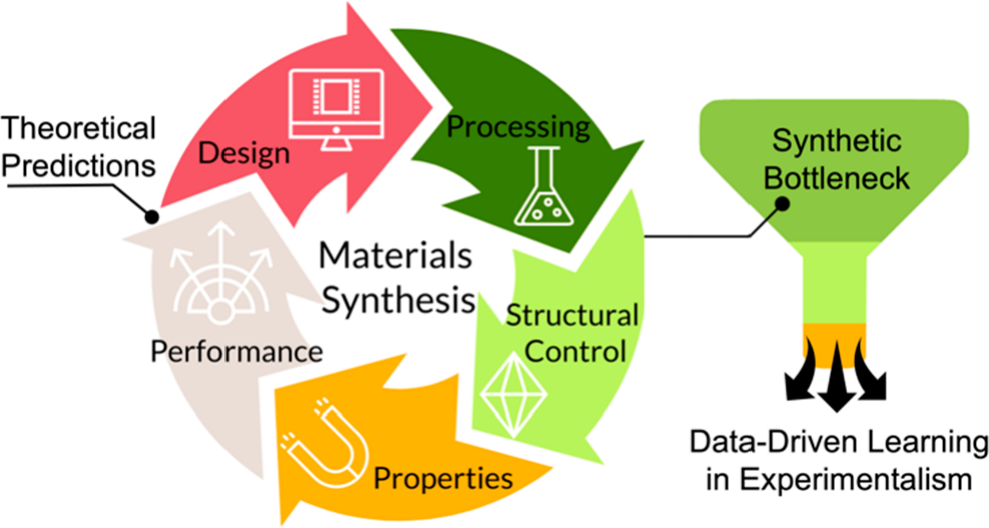

The design of inorganic materials for various applications critically depends on our ability to manipulate their synthesis in a rational, robust, and controllable fashion. Different from the conventional trial-and-error approach, data-driven techniques such as the design of experiments (DoE) and machine learning are an effective and more efficient way to predictably control materials synthesis. Here, we present a Viewpoint on recent progress in leveraging such techniques for predicting and controlling the outcomes of inorganic materials synthesis. We first compare how the design choice (statistical DoE vs machine learning) affects the type of control it can offer over the resulting product attributes, information elucidated, and experimental cost. These attributes are supported by discussing select case studies from the recent literature that highlight the power of these techniques for materials synthesis. The influence of experimental bias is next discussed, followed finally by our perspectives on the major challenges in the widespread implementation of predictable and controllable materials synthesis using data-driven techniques.

## Abstract 2

The design of inorganic materials for various applications critically depends on our ability to manipulate their synthesis in a rational, robust, and controllable fashion. This Viewpoint presents the current progress in data-driven techniques as a more effective and efficient way to accomplish such control.

## Introduction

Every example of modern technology that is enabled by inorganic materials is also limited by inorganic materials; for example, the various physicochemical properties of inorganic materials limit the conversion of sunlight into electricity or fuel, limit our ability to efficiently catalyze chemical reactions under mild conditions, and limit how we generate artificial light.^1^ The search for new inorganic materials is driven by the need to improve these existing technologies, in addition to the discovery of novel functionality to realize the next generation of new technologies. As such, there is a need to design, discover, and synthesize novel materials. This is essential for scientific progress and long-term economic growth.^1^

**Scheme 1:**
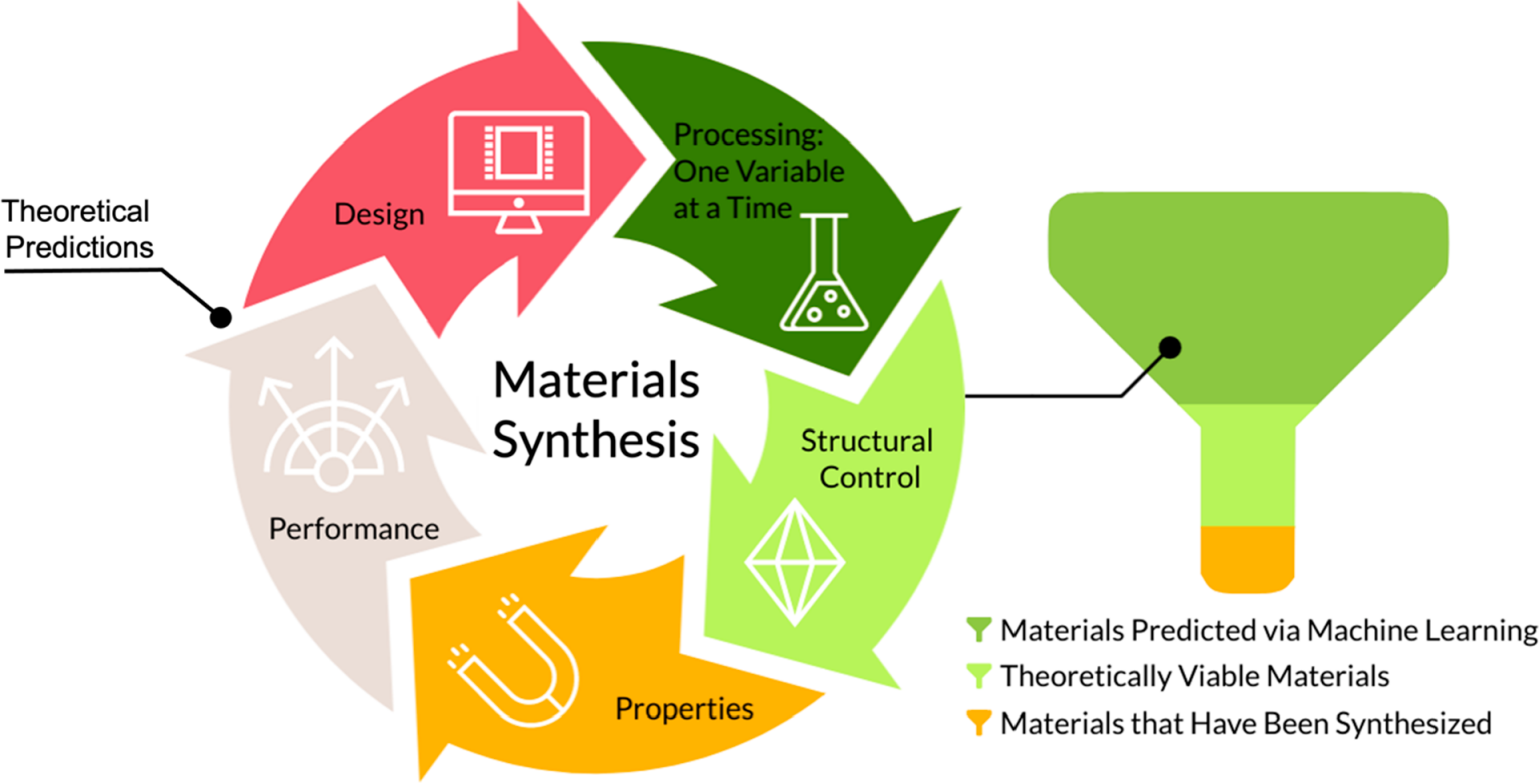
Idealized Process of Materials Predictions, Engineering, and Synthesis

Not only do the scale and urgency of these materials require the discovery of next-generation materials, but also these materials must then be made and deployed at a much more accelerated pace than the historical multidecadal gap between discovery and commercialization.^2^ On the front end, materials informatics and machine-learning-based computational discovery via the Materials Genome Initiative has greatly accelerated materials discovery through efforts such as the Materials Project, which combines supercomputing and density functional theory to predict materials and their properties before they are made.^3−5^ While this “materials by design” approach, developed over a decade ago, has successfully identified vast numbers of materials with a wide range of targeted properties, a significant bottleneck now exists in the next step of the process, which is the actual synthesis of these predicted materials (Scheme 1).

The research and development of inorganic materials critically depend on our ability to manipulate their synthesis in a rational, robust, and controllable fashion. Despite this, in the attempt to synthesize both known and unknown materials, most materials synthesis is not based on a deep mechanistic understanding. This complicates prediction and control. Precise synthetic control is further complicated by potentially complex experimental parameter spaces, which can be large and have high dimensions. Regardless of the capability of machine learning to suggest new families of materials with advantageous properties, laboratory synthesis and optimization depend on the careful consideration of experimental parameters like reagent choice, synthetic method, temperature, reaction time, atmosphere and pressure, stoichiometric ratios, concentrations, solvents, additives, etc.^1^ This convolutes chemical intuition because understanding how the various experimental parameters interact to affect the synthetic outcomes can be highly complex. Additionally, the compositions and structure types of crystalline inorganic solids are so disparate that it is exceptionally challenging to apply the lessons learned from one synthetic system to another. In fact, even “simple” binary systems can exhibit thermodynamic phase diagrams that are synthetically challenging to navigate.

As a result, most materials synthesis is Edisonian in nature, where one variable is adjusted at a time to assess the outcome. This one-dimensional technique, often called one-variable-at-a-time (or OVAT) is slow and random. Advances in new combinatorial synthesis methods, like parallel syntheses via scanning probe lithography or multiple partial cation exchange reactions on metal chalcogenide nanocrystals, can accelerate materials discovery by producing larger libraries with increasing volumes of data; however, these techniques are generally low throughput in terms of the absolute amounts of material produced.^6,7^ Alternatively, methods that aim to increase the synthetic throughput of materials, like the use of parallel-flow reactors, still face the same one-dimensional challenges as batch syntheses.^8^ It can take years for the successful design, execution, and optimization of a materials synthesis, which has not advanced as far or as fast as computational predictions. In other words, synthesis has been overshadowed and overwhelmed by the rapid success and growth of predictive simulation.^9^ This is due, largely in part, to the lack of robust predictive synthetic frameworks that can help map or guide a reaction coordinate from reagents through nucleation and growth to the final crystalline solid. The inefficiencies of OVAT methods render the ascertainment of a full picture of any particular materials system realistically unachievable, and true optima are therefore rarely identified.^2^ Consequently, materials discovery and optimization for target applications require the adoption of more advanced techniques to better match the pace of data-driven material predictions.

A solution to this problem lies at the interface between disciplines. Implementing data-driven techniques at the experimental end of materials synthesis and optimization is a tractable and natural progression of materials informatics. In this perspective, we describe how proper utilization of such techniques can dramatically increase the rate and efficiency of experimental screening and optimization in inorganic materials chemistry. Specifically, statistics-based regression techniques like the design of experiments (DoE) and response surface methodology (RSM) enable multivariate investigations of the experimental domain for a specific material system, which can map synthetic outcomes that are continuous in nature (i.e., emission wavelength, yield, surface-area-to-volume ratio) as a function of input variables. This can provide key mechanistic insight and a multidimensional view of the experimental design space from only a fraction of the experimental observations typically needed for OVAT methods. Machine learning can similarly be utilized; that is, machine-learning classifiers enable the mapping of categorical (or discrete) synthetic outcomes like the crystal phase. Neural networks and iterative active learning can uncover complex synthesis–structure–property connections that human intuition cannot. Herein, we discuss the advantages, challenges, and future directions of leveraging data-driven techniques to advance the synthesis and optimization of inorganic materials and critically assess recent developments.

## DoE versus Machine Learning

An important step toward the implementation of data-driven techniques is determining which techniques best solve specific materials synthesis problems. To address this, we will first assess the information afforded via DoE and the subsequent analysis. We will then similarly compare the information afforded via machine-learning techniques.

### Advantages of DoE

The basic ideas behind statistical modeling and DoE date back to as early as 1935.^10^ DoE has since demonstrated its utility in industry, including manufacturing and engineering,^11−18^ and its application to problems in chemistry has seen a rapid increase in the last two decades.^19,20^ Particularly, there has been a sharp rise in examples of its utility in optimizing materials syntheses in academic literature, including a recent tutorial outlining the use of DoE for materials chemistry.^1,2,21−34^ The increased use of DoE can be attributed to its effectiveness at achieving synthetic control of both dependent and independent variables when used in conjunction with RSM, which is an optimization technique that provides insight throughout a high-dimensional parameter space to formulate a predictive model by using a minimal number of experiments.

The ability to maximize the information that can be extracted from small data sets makes DoE ideal for novel, low-throughput, and/or exploratory materials synthesis where little is known about the system at hand or the ability to collect large amounts of data is limited. Several variables can be screened at once in a rational manner using tailored screening designs, which allows a broad, multivariate parameter space to be sampled quickly and systematically in a minimum number of experiments with the ability to assess different variables with varying degrees of importance or weights. In the choice of variables in the experimental design, categorical variables like reagent type require an exponential increase in experimental observations compared to continuous variables, so care must be taken to ensure the most beneficial design. Subsequent analyses are regression-based, making DoE investigations mainly suitable for synthetic outcomes that are continuous in nature, such as the emission wavelength or yield. This favors the use of DoE within the parameter space for a specific material crystal phase and not mixed-phase systems.

**Figure 1:**
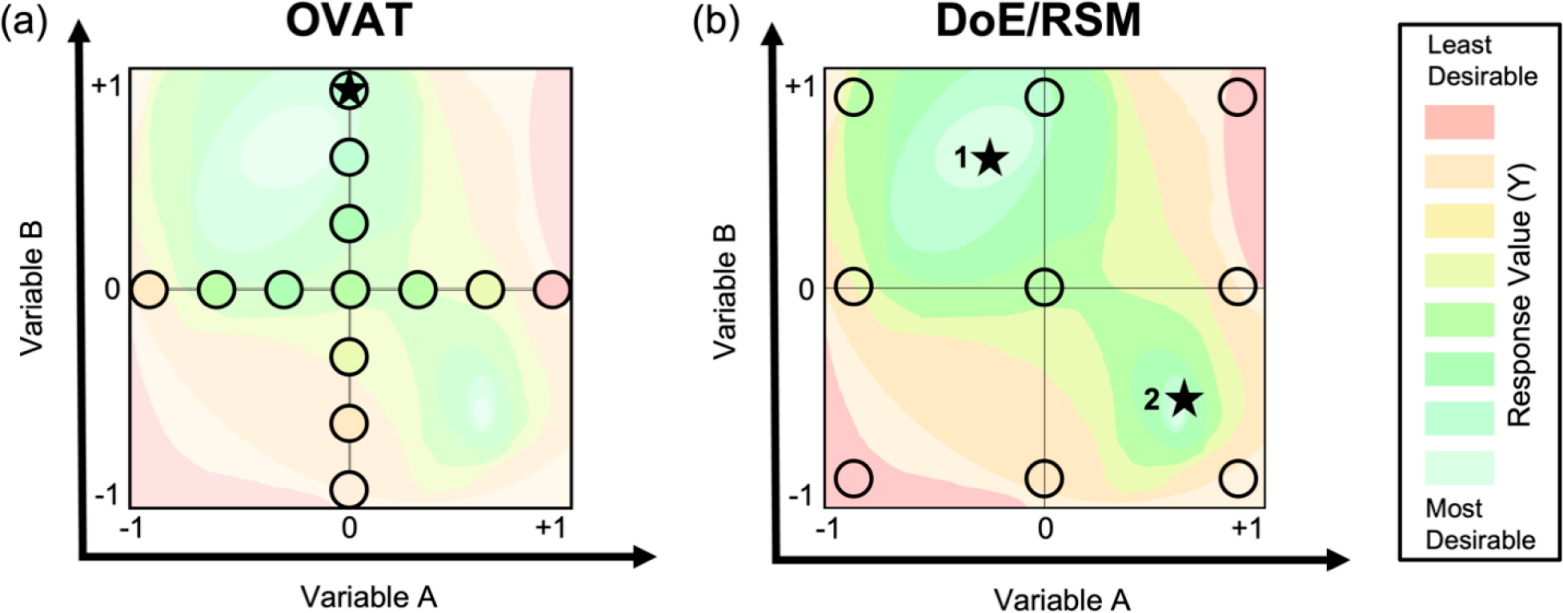
Visual example of (a) the OVAT method and the information elucidated within a two-variable parameter space versus (b) the information elucidated throughout the same two-variable parameter space using the combined DoE/RSM method. Circles represent the experiments performed within the parameter space. Stars represent the optimal values found by each respective method. Adapted with permission from ref (2). Copyright 2022 American Chemical Society.

After the experiments indicated by a given experimental screening design are performed, statistical analyses of the obtained experimental data can uncover which variables have statistically significant effects on the assessed outcomes. This includes the roles of higher-order interactions between two or more variables and how they dictate the characteristics of the product, which are not possible using traditional OVAT techniques and are often beyond the reach of human intuition. The acquired data can then be used to fit either a linear (first-order) or polynomial (higher-order) model to create a *response surface* of the different product characteristics as a function of the variables screened using RSM. These multivariate models quickly identify a set of experimental conditions that lie at the minimum or maximum or hit a set target within the response surface parameter space, which typically would be missed using one-dimensional experiments (Figure 1). This easily facilitates the optimization of a material product, affording essential control over application-specific characteristics like particle size, yield, band gap, power conversion efficiency, thin film thickness, emission wavelength, etc. With that being said, not all materials synthesis problems are optimization problems, for which the DoE sampling techniques are often readily amenable to modeling with machine-learning algorithms.^35^

### Advantages of Machine Learning

The implementation of machine-learning techniques in materials chemistry is even more recent, with the majority of its use in academia only coming in recent years.^35−41^ Despite machine-learning techniques requiring notoriously large data sets, which can be limiting in the field of exploratory materials synthesis, the raw computing power renders it attractive for mapping complex synthesis–structure–property relationships that are unachievable with traditional approaches. When coupled with new advancements in self-driving, high-throughput synthetic techniques such as multichannel flow reactors or automated robotic systems, which permit a larger number of experimental observations to be collected in a shorter amount of time, the opportunities for systematic exploration of synthetic landscapes are greatly expanded.^36,42−45^

**Scheme 2:**
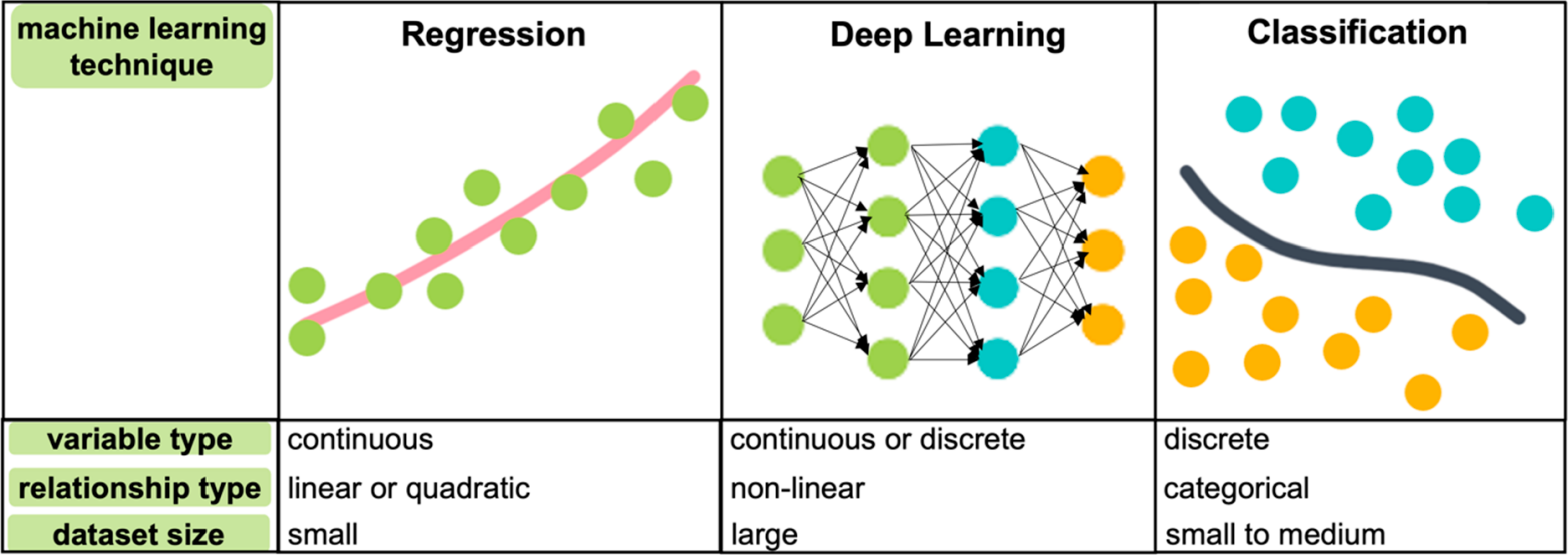
Basic Machine-Learning Techniques for Different Response Types and Complexities

When dealing with more complex materials systems where design space exploration, rather than optimization, is the focus, machine learning is a more appropriate option.^46^ As opposed to the DoE, machine-learning techniques can handle both mixed and categorical variables and synthesis outcomes (Scheme 2). This renders them better suited for problems involving the synthesis of new materials across a wide range of crystal phases; for example, Tamura et al. recently considered strategies to effectively construct phase diagrams using high-throughput batch experiments.^47^ For synthetic problems where high-throughput synthesis is not plausible, data-driven classifiers can train on relatively small data sets to render synthetic phase maps that allow for the rational targeting of materials within a high-dimensional parameter space.^48^ The efficacy of the experimental training data sets can be maximized by utilizing design matrixes typically seen in the DoE to rationally sample an *n*-dimensional design space and increase its robustness. This provides the classification algorithm with the training and testing data required to map the patterns of the experimental variables, identify the roles of variables in the synthesis, and determine which variables are the most important for the determination of a discrete outcome. Analysis of the trained models and their decision-making process can provide vital chemical insight, offering a route for prescriptive materials outcomes.

For problems involving a fundamental lack of chemical understanding, deep learning techniques like neural networks can elucidate complex synthesis–structure–property relationships but typically only when provided with large data sets.^39,49,50^ Consequently, their implementation is more appropriate when used in conjunction with high-throughput experimentation techniques or situations where the literature can be extensively datamined. This often renders them unsuitable for problems involving novel or inherently low-throughput chemistry. Alternatively, iterative or active learning techniques using methods like Bayesian optimization can mitigate the downfalls of deep learning and deal with smaller data sets.^51−60^ By balancing the exploitation of known data with the exploration of unknown parameter spaces, they are good alternatives for mixed-variable optimizations that are not as straightforward or plausible with DoE.^52,53,55,61,62^

## Case Studies

### Examples of DoE Applied to Materials Synthesis

**Scheme 3:**
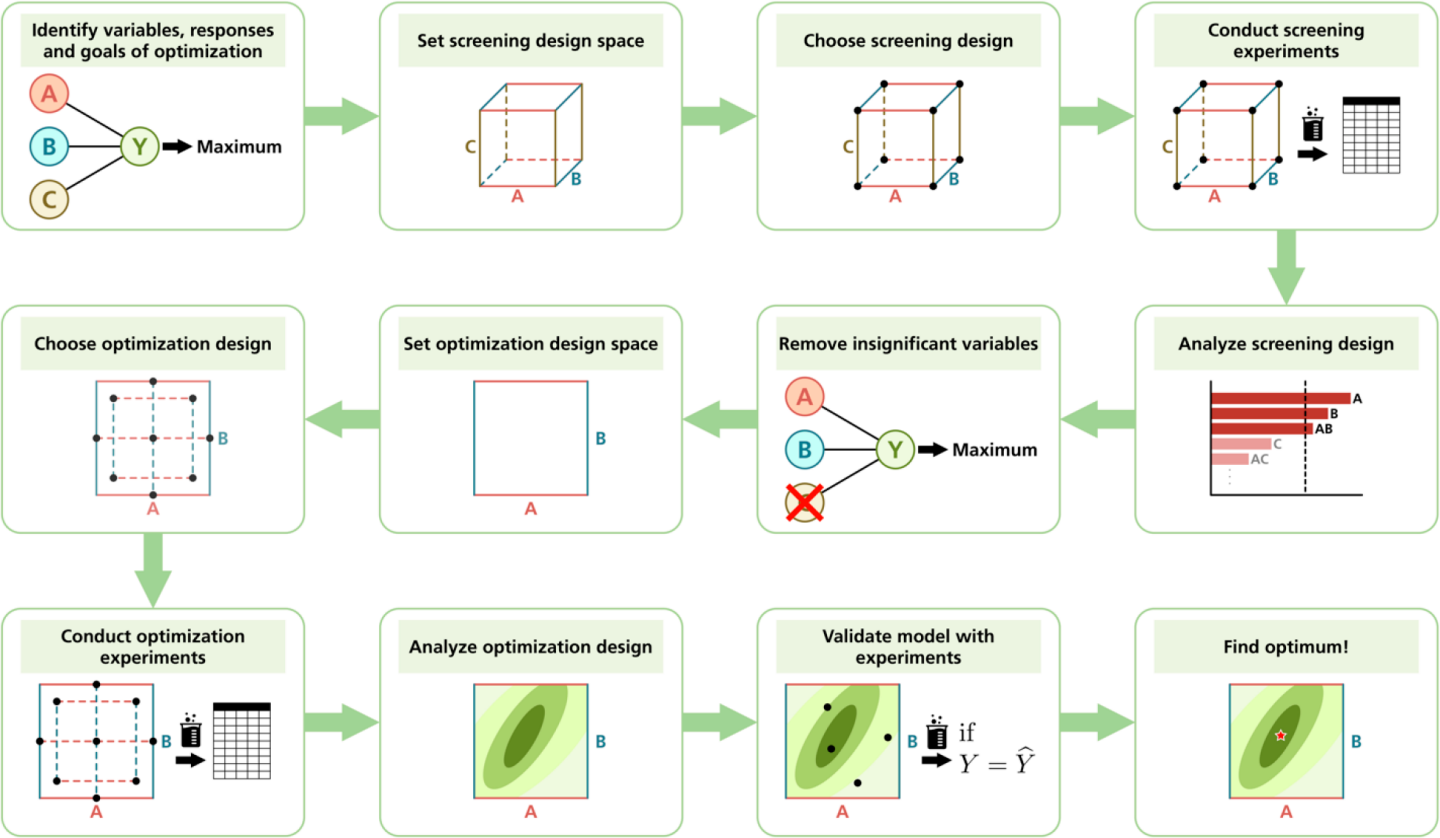
Flow Chart of DoE Implementation to Optimize a Material Synthesis, Exemplifying an Eight-Experiment Screening of Three Factors with a Subsequent Second-Order Optimization Adapted with permission from ref (2). Copyright 2022 American Chemical Society.

There are numerous examples in the literature illustrating the use of DoE techniques across a wide range of problems in inorganic materials synthesis. In such DoE studies, after the research goals are defined, the initial round of sampling is often used to down-select the variables being screened through the feature selection of those with the greatest influence on synthetic outcomes (Scheme 3). This allows a second round of more dense sampling of the design space to be performed. RSM utilizes the steepest-ascent approach, which involves iterative sampling in the direction that heads toward an optimum output.^35^ While the data are ascending, a first-order, linear model is used that does not account for curvature in the output data. Once near the apex in the data, the second round of sampling enables an assessment of second-order variable influences, or curvatures in the response surface, yielding a quadratic model that provides a better fit.^2^

**Figure 2:**
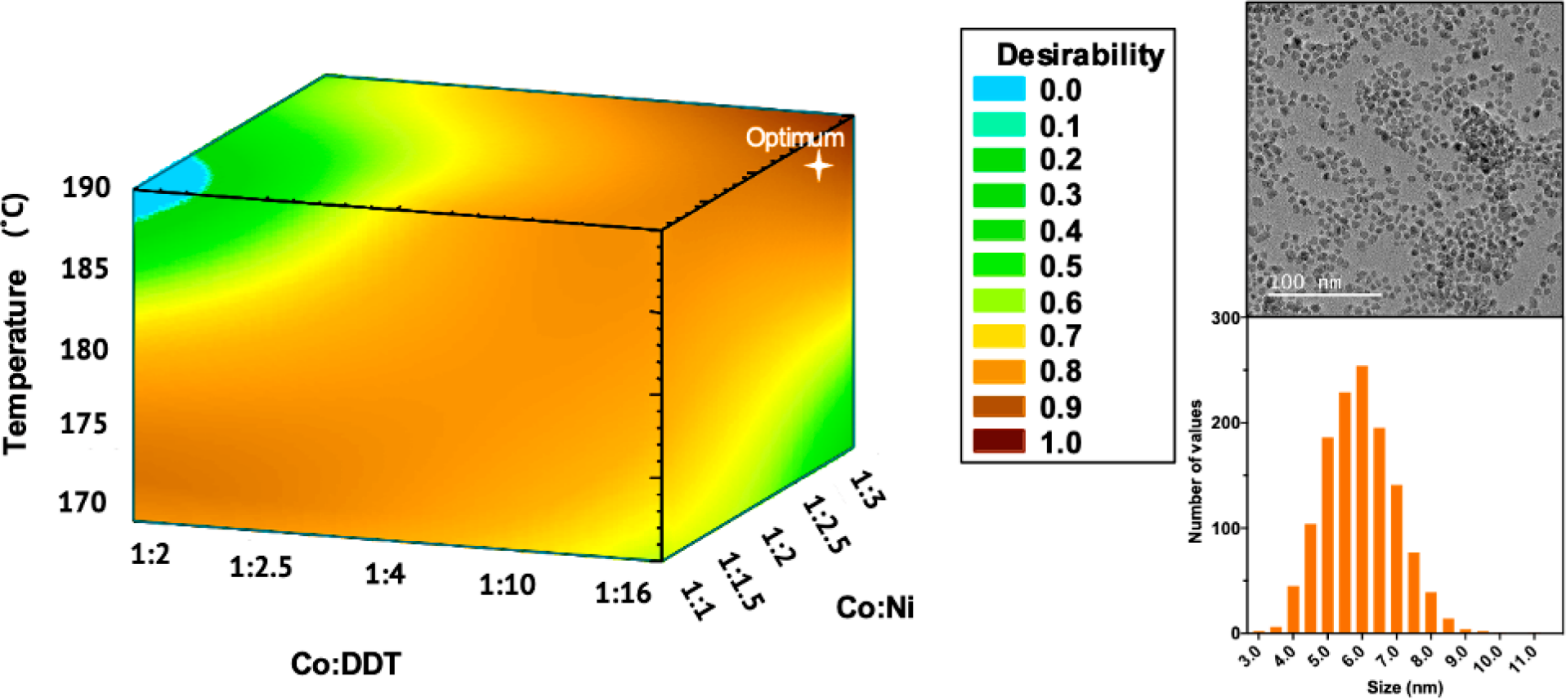
(Left) Desirability plot of the simultaneous optimization of all three parameters (minimizing the nanocrystal size and polydispersity, maximizing the isolated yield), with the global optimum indicated in white. (Right) Transimission electron microscopy image of the CoNi_2_S_4_ nanocrystals synthesized under the optimal conditions and the corresponding size histogram of the resulting nanocrystals, where σ/*d̅* = 14% (*N* = 1300). Adapted with permission from ref (27). Copyright 2021 American Chemical Society.

DoE can be utilized for a range of scientific inquiries, and several successful examples have been published with a focus on materials synthesis, demonstrating the ability of DoE to successfully analyze the trends, magnitude of influence, and correlation of large sets of synthetic variables. For example, Williamson et al.^27^ performed a multivariate optimization of the colloidal synthesis of CoNi_2_S_4_ inverse thiospinel nanocrystals to produce a small, monodisperse, and nanocrystalline product in high yield. In the first-order design, the influences of five experimental variables were screened to assess their influence and generate a model predicting the nanocrystal size, polydispersity, and isolated yield. Only three of the five variables (i.e., Co:Ni ratio, Co:S ratio, and reaction temperature) had statistically significant effects, so a second-order design was then performed to model the parameter space at a higher number of levels for these three variables. This model was jointly optimized by using a desirability function to find the global optimum (over all three responses) within the parameter space, which predicted the set of conditions to produce the most optimal product (Figure 2). This is sometimes referred to as a “Pareto frontier” optimization. Figure 2 defines the Pareto frontier, or a graph that plots a set of solutions that represent the best trade-off between all of the objective functions (in this case, there are three).^63^ The prediction was validated experimentally, and the results were in excellent agreement with the predicted values, highlighting the ability of the DoE to efficiently traverse unknown parameter spaces to predict the optimal synthetic conditions.

**Figure 3:**
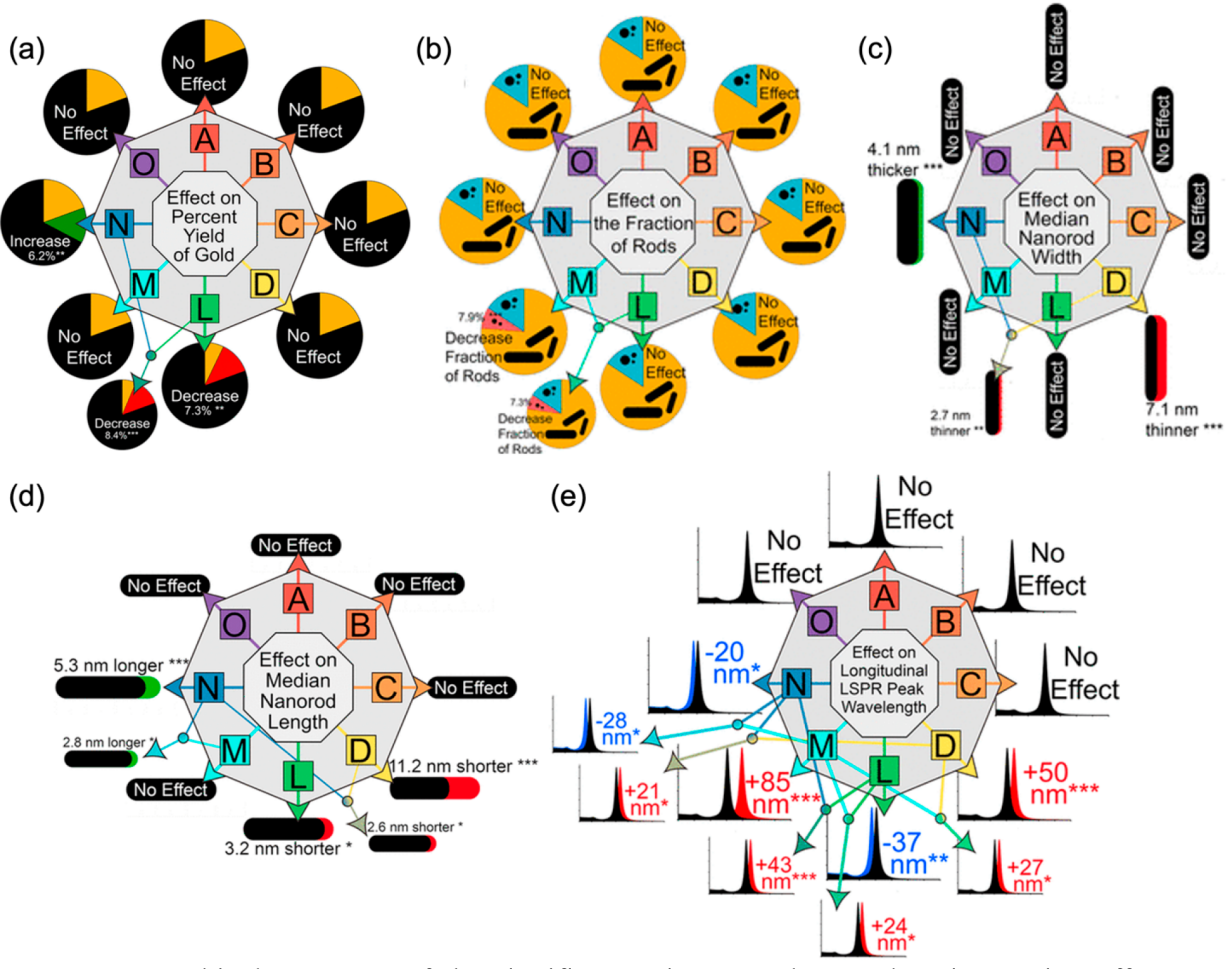
Graphical summary of the significant primary and secondary interaction effects on (a) the percent yield of gold nanorods, (b) the fraction of rods to shape impurities, (c) the median rod width, (d) the median rod length, and (e) the longitudinal surface plasmon resonance peak energy. Each outcome represents 100% of the response, where the gold (a and b) or black (c–e) illustrates the average response, and the colors represent an average increase/decrease of the reported response. All effects are reported as a response to increasing factor levels, where *A* = amount of NaBH_4_, *B* = rate of stirring of the seed solution, *C* = age of the seed solution, *D* = number of seeds, *L* = temperature, *M* = amount of silver promoter, *N* = amount of ascorbic acid, and *O* = age of the reduced solution. *P* values: (***) < 0.001 < (**) < 0.01 < (*) < 0.05 < (.) < 0.1 < () < 1. Adapted with permission from ref (22). Copyright 2017 American Chemical Society.

In addition to determining which factors are important, the DoE can provide detailed insight by measuring the degree to which variables interact with each other. For example, Burrows et al.^22^ used a composite fractional factorial series of experiments to address common challenges in the seeded growth synthesis of gold nanorods.^64−67^ The experimental design screened eight potential variables to control the gold nanorod synthesis (*A* = amount of NaBH_4_, *B* = rate of stirring seed solution, *C* = age of seed solution, *D* = number of seeds, *L* = temperature, *M* = amount of silver promoter, *N* = amount of ascorbic acid, and *O* = age of reduced solution) and their effects on five different responses: the effect on the percent yield of gold nanorods, the effect on the fraction of rods, the effect on the median nanorod width, the effect on the medium nanorod length, and the effect on the longitudinal surface plasmon resonance peak wavelength (Figure 3). For example, looking at the output of the DoE results for the percent yield of gold nanorods in Figure 3a, the temperature (*L*) resulted in a 7.3% decrease in the percent yield of gold nanorods when temperature was increased from low to high levels. Similarly, the interaction between the temperature and amount of silver promoter (L and M) resulted in an 8.4% decrease in the percent yield of gold nanorods in response to increasing both variables from the low to high levels, whereas the silver promoter (M) alone had no statistically relevant effect on the yield. In contrast, increasing the amount of ascorbic acid (*N*) resulted in a 6.2% increase in the percent yield of gold nanorods. All other variables (*O*, *A*, *B*, *C*, and *D*) had no significant effect. After the results of the other four responses were analyzed in a similar manner (Figure 3b–e), mechanistic conclusions could be drawn. The authors were able to assign the basic mechanism of gold nanorod formation to the reduction of auric ions by ascorbic acid onto seed nanoparticles. This subsequently elucidated a route to a more reproducible and robust nanorod synthesis, concluding that intentional control of the amount of ascorbic acid, the reaction temperature, and especially the number of seeds added affords the most optimal synthetic control. The effects of these eight synthetic variables on five responses using this technique offered a 40-fold (8 × 5) increase in the magnitude of progress afforded in a single study compared to what is possible using common OVAT techniques, exemplified by both key mechanistic insights and a synthetic optimization of gold nanorods.

DoE has also been applied to large data sets to directly screen data libraries for possible next-generation materials properties. For example, Maier et al. screened the catalytic performance of 467 different materials for propene oxidation.^68^ Dellamorte et al. adopted DoE for finding a suitable bimetallic catalyst for ethylene epoxidation.^69^ Corma et al. combined DoE with high-throughput experimental screening and data mining to develop new catalytic zeolite materials, monitoring the crystallinity of zeolites as a function of the synthesis conditions (i.e., using the molar ratios of the educts as input factors). This enabled the discovery of a new catalytically active and selective zeolite (ITQ-30) for the alkylation of benzene with propene.^70^ These examples exemplify the ability of DoE to provide the tools to reopen the synthetic bottleneck of materials discovery, in addition to fine-tuning and optimizing the materials properties and reaction conditions. The combination of high-throughput experimentation with DoE methods, like those used by Corma et al., has also been extended to other materials systems. Reinhardt and co-workers^71^ used the well-studied lead halide perovskite MAPbI_3_ (MA = methylammonium) as a model system to establish an automated routine for the rapid identification of conditions for optimal film deposition. In their work, they provided a direct comparison between the manual optimization protocols and high-throughput experimentation, combined with the DoE statistical analysis. This work highlighted that automation and peak ratio analysis outperformed manual techniques by only requiring about 60% of the experimental time. This shows promise for the combination of high-throughput techniques with DoE; however, for a larger materials space, such as the screening of vast compositional libraries for new materials compositions, machine learning would be the technique of choice over DoE, as discussed below.

### Examples of Machine Learning Applied to Materials Synthesis

**Figure 4:**
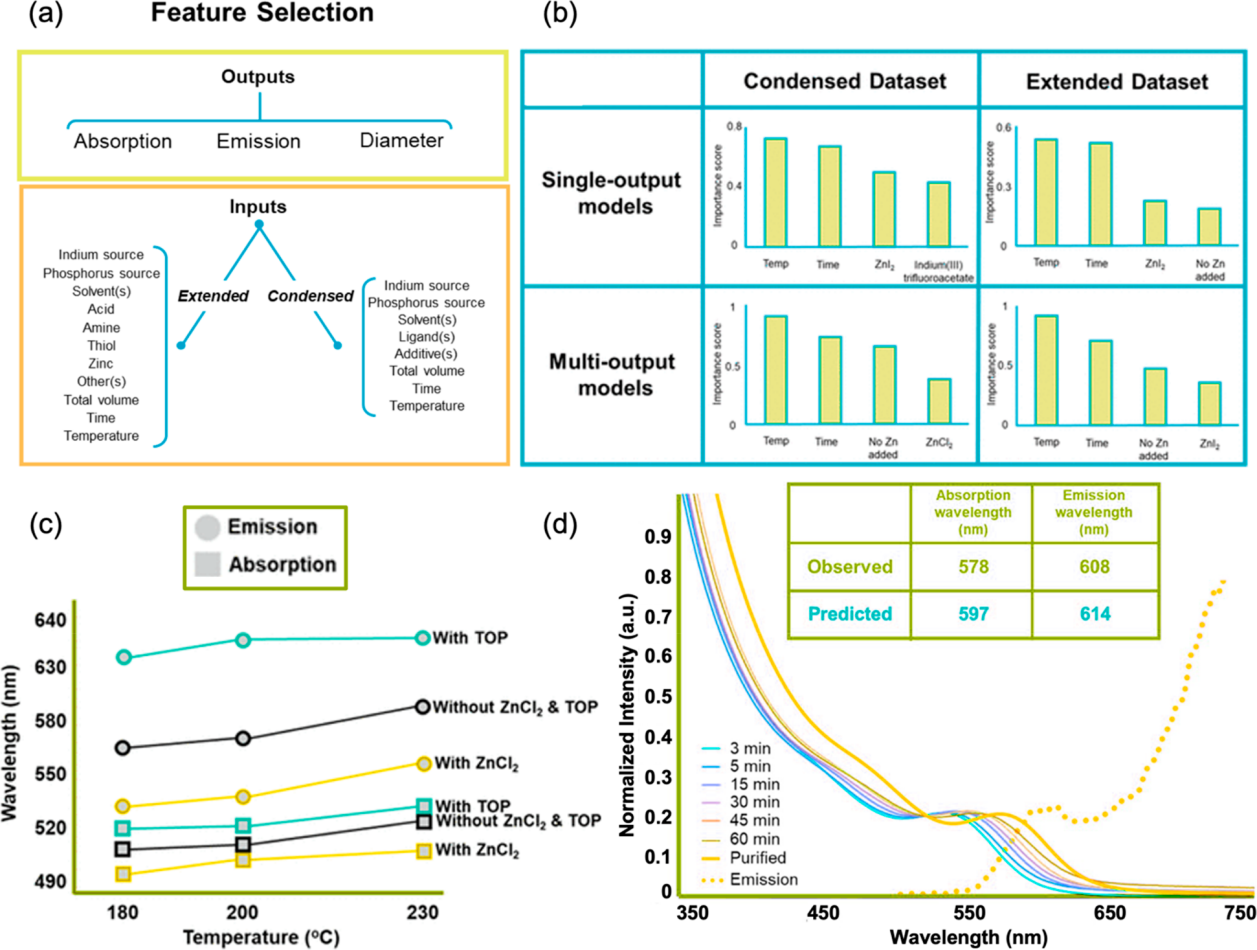
(a) Output targets for the synthesis of InP quantum dots and the synthetic parameters of each data set. (b) Feature importance charts for the best model in each study case. (c) Predicted emission (circles) and absorption (squares) wavelengths from the Streamlit web app using single-output algorithms and the condensed data set with all methods. (d) Observed and predicted absorption wavelengths, emission wavelength, UV–vis spectra of timed aliquots, and emission spectrum of the purified product from the reaction predicted by the web app. Adapted with permission from ref (73). Copyright 2022 American Chemical Society.

While there is no question that DoE/RSM provides a distinct advantage over OVAT sampling and allows for reliable inferences from a small amount of data, machine-learning methods can provide valuable insight for more complex responses and develop useful models that can be iteratively improved. In general, as previously mentioned, machine-learning techniques are particularly well suited to large amounts of data with complex prediction- or optimization-based goals. While such goals remain the same for materials synthesis, the cost of data is much higher.^72^ The sparsity of data sets and the substantial costs of experiments have limited its application in the exploration of synthetic design spaces,^35^ but for those syntheses with abundant data, machine-learning algorithms can effectively generate models despite the random distribution of data points. For example, the recent work of Nguyen et al. extracted data from 72 publications to define a set of extended and condensed synthetic conditions to determine the best approach for predicting synthesis outcomes where the available data are limited (Figure 4a).^73^ Both condensed and extended descriptor spaces were used to train different models to predict three relevant properties of InP quantum dots as output targets: the quantum-dot diameter, the absorption edge energy, and the photoluminescence emission energy. After training, testing, and analyzing the model estimation errors of single-output and multioutput machine-learning algorithms, the authors concluded that the reaction temperature, time, and addition of zinc salts had the greatest impact on the InP quantum dot properties (Figure 4b).

Single-output models using Extra Trees and Decision Tree algorithms trained on the condensed data set showed the best predictability, despite the physical relationships among the output targets. Thus, these models were employed in a web app called Streamlit that enabled real-time reaction analysis and prediction, allowing an exploration of the chemical intuition of the algorithms beyond statistical metrics and the discovery of synthetic trends without conducting actual experiments. For example, predicted outcomes from the web apps suggested that, for a typical hot-injection synthesis where InCl_3_ reacts with tris(diethylamino)phosphine, the presence of trioctylphosphine (TOP) redshifts the emission and absorption maxima, while the presence of a zinc halide salt results in spectral blueshifts (Figure 4c). These observations are consistent with those reported in the reported literature. Additionally, the authors were able to target ∼600 nm absorbing InP quantum dots using synthetic conditions and precursors from an existing procedure, with the reaction conditions predicted by the web app resulting in InP quantum dots with the desired optical properties (Figure 4d). This work provides a procedure to preprocess data sets, train machine-learning models, and implement models for public users in the field of colloidal nanocrystal synthesis, especially where available data sets are small and incomplete. It is a step in the right direction for the application of machine-learning algorithms to reshape how scientists elucidate chemical and synthetic insights. Despite this, after entering synthetic conditions into the web app to obtain predicted values and comparing them with experimentally measured values, the mean absolute errors on the experimental values were generally higher than the values from the test sets due to the small size of the experimental set, thus showing that such algorithms cannot recognize new precursors, and moving forward, the reaction conditions need to be closely based on existing procedures to obtain accurate predictions.

When a large data set is not available, the support vector machine (SVM) algorithms can provide more efficient sampling within the design space compared to DoE and capture nonlinear relationships from limited data. Braham et al. utilized SVM models to predict the thickness of quantum-confined CsPbBr_3_ nanoplatelets based on the reaction conditions from a relatively sparse data set.^74^ The initial step involved the use of a classification model to divide the design space into two distinct regions, yielding quantum-confined and bulk products, respectively. Then a regression analysis was conducted within the quantum-confined region for the prediction of the layer thickness based on the reaction temperature, concentration, and diffusion coefficient of alkylamine ligands. This study not only enabled CsPbBr_3_ nanoplatelet synthesis with monolayer precision but also provided insights into the growth mechanism, showing that it is possible to achieve accuracy with small data sets and machine-learning algorithms. Furthermore, the combination of SVM classification and regression provides a broadly generalizable means of developing robust predictive models from limited data sets, but better numerical descriptors need to be developed as an initial step.

**Figure 5:**
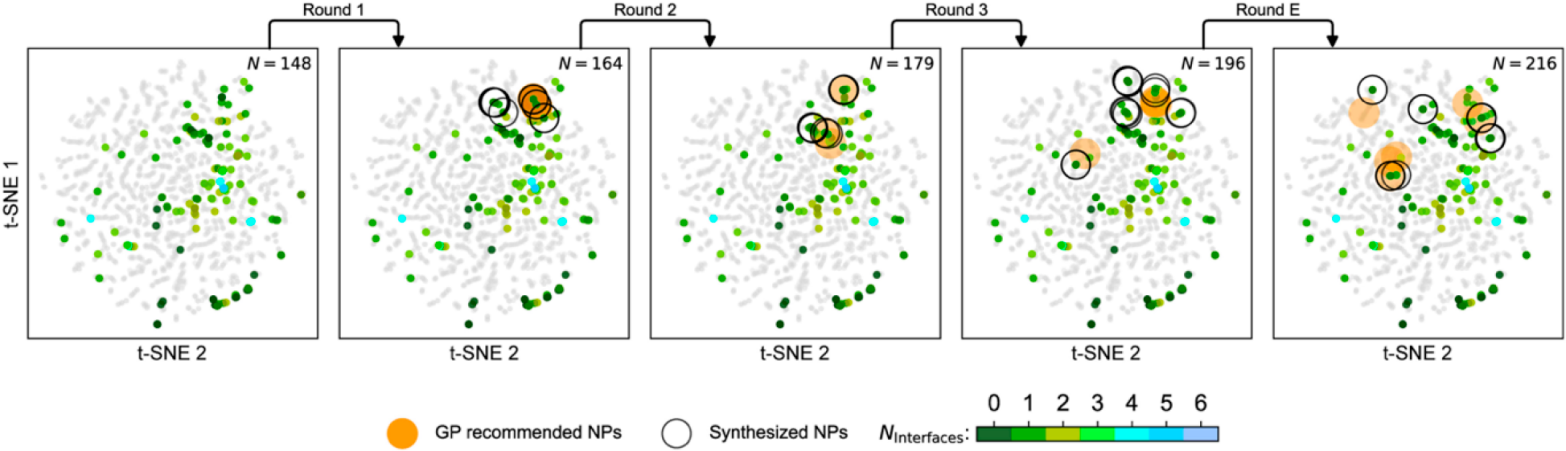
Characteristics of an 8D nanocrystal space and its Bayesian-guided exploration shown via a 2D projection of the Bayesian optimization design space. Gray circles represent the entire compositional search space of the 8D compositional grid. The experimental data (both initial and acquired in the loop) are color-coded with measured interface counts (*N*_interface_). Acquisition suggestions by the algorithm (large open circles) and synthesized suggestions (large blue circles) are overlaid on the same projection to provide visual insights to how the search space is traversed with the aid of the algorithm. Round E corresponds to the “exploratory” search toward modified targets carried out upon completion of the closed-loop iteration. Adapted with permission from ref (75). Copyright 2021 Science Advances.

Another suitable option for smaller data sets is iterative optimizations that utilize active learning. Bayesian optimization algorithms can receive feedback from experiments in an iterative manner to guide the exploration of the design space. A visualization of this type of iterative exploration of a parameter is given in Figure 5, which exemplifies a Bayesian-guided exploration of an eight-dimensional (8D) parameter space for the discovery of polyelemental heterostructured nanocrystals by Wahl et al. They utilized active learning with Bayesian optimization algorithms to explore the synthesis of heterostructured nanocrystals with novel compositions and certain interfacial motifs,^75^ where scanning transmission electron microscopy–energy-dispersive X-ray data including the elemental compositions and number of interfaces were fed into the algorithm after each experiment. This approach resulted in the synthesis of quaternary to senary biphasic heterostructures with a high elemental complexity and potentially superior catalytic properties. The authors demonstrated that machine-learning-assisted optimization is a viable approach to identifying nanostructures with target morphological qualities, even with limited data sets, as this platform enabled the synthesis of new and complex materials, including the purported most complex biphasic nanocrystal ever made, with success rates (95%) significantly better than random selection. The limitations for expanding such a design feedback loop to higher-throughput synthesis and experimentation and into more complex properties, such as catalytic activity or stability, are almost exclusively experimental, illustrating the need for new automated characterization techniques to extract more information from the megalibraries of nanomaterials at much higher throughputs.

**Figure 6:**
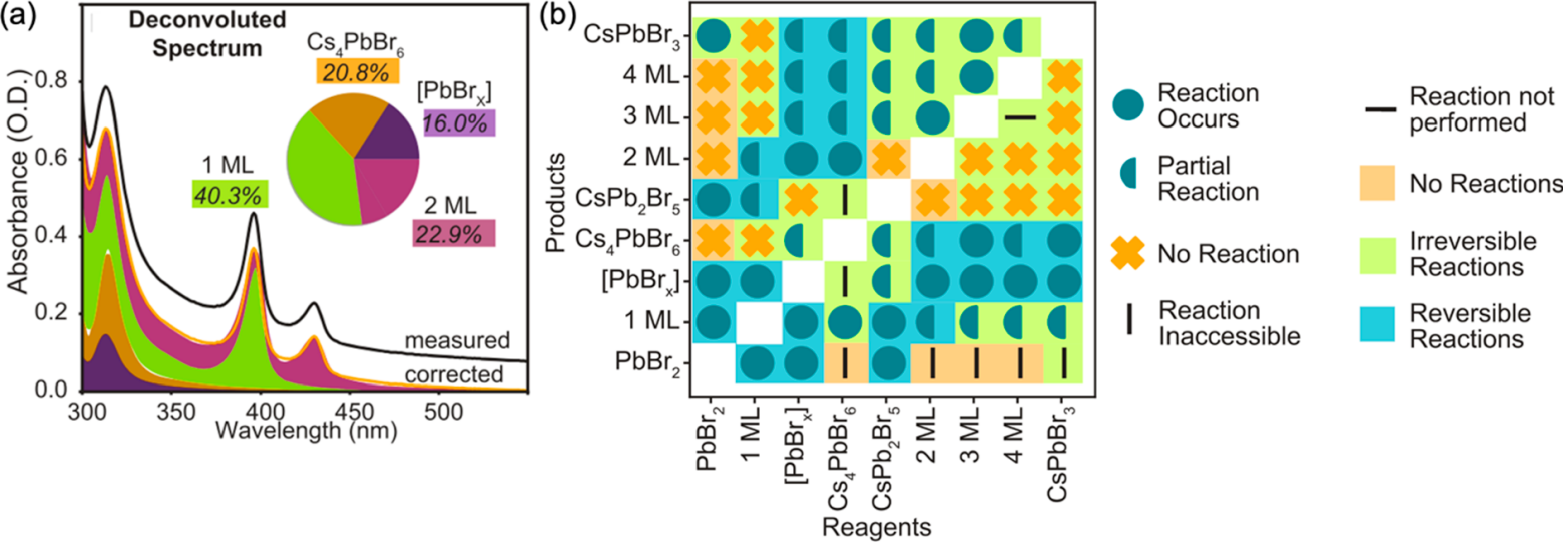
(a) Deconvoluted example spectra showing the fractional contributions of [PbBr_*x*_], Cs_4_PbBr_6_, and 1 and 2 monolayer nanoplatelets. (b) Matrix classifying all pairs of transformations by the observed outcome of a Cs–Pb–Br compositional space. Inaccessible reactions are transformations in which there is no possible single-reagent route from the reagent species to the product species in the parameter space. Reversible reactions correspond to both possible reactions being observed, and irreversible reactions correspond to one of the two possible reactions observed. Adapted with permission from ref (76). Copyright 2020 American Chemical Society.

Besides predicting the outcomes of materials syntheses, machine-learning techniques have also proven to be useful for batch-processing the experimental data. For example, Dahl et al. developed a phase map of the Cs–Pb–Br perovskite nanocrystal synthesis from 2337 reactions using a high-throughput liquid handling robot, which added dodecane, oleylamine, lead oleate, oleic acid, and cesium oleate in varying proportions to a 96-well plate to react and subsequently obtained optical spectra.^76^ Spectral characterization was accomplished in a high-throughput manner via a microplate reader, followed by the implementation of a least absolute shrinkage and selection operator with a cross-validation (LASSO-CV) algorithm, which automatically deconvoluted the UV–vis absorption spectra into ratios of various components (Figure 6a). The collective UV–vis data were deconvoluted among 11 unique product species to produce phase maps. Using the region boundaries of the phase maps, a reaction network was proposed that revealed the equilibria and interconversion among various molecular complexes, monolayered platelets, and nanocrystals (Figure 6b). By combining factorial exploration of the synthesis space and an automated method for deconvoluting absorption spectra to create maps and transformations, the authors showed how both quantitative and qualitative analyses of high-throughput synthesis data can determine a cohesive chemical reaction network for a complex compositional space.

## Challenges and Future Directions

Both statistical techniques and machine learning can accelerate our understanding of materials design, synthesis, and discovery. The examples given in this Viewpoint show that these approaches have the capability of being transferred to other complex inorganic systems for a range of problems. DoE addresses several of the problems for novel, low-throughput, and/or exploratory materials synthesis, where little is known about the system at hand or the ability to collect large amounts of data is limited by its ability to maximize the information that can be extracted from small data sets using multivariate screening techniques. When dealing with more complex materials systems where design space exploration, rather than optimization, is the focus, machine learning is a more appropriate option. The raw computing power renders it attractive for mapping complex synthesis–structure–property relationships that are unachievable using traditional approaches. Both techniques offer a more unsupervised route to materials synthesis by removing a large portion of manual inputs from the experimenter, which inherently alleviates problems with experimental bias and expedites the synthesis of novel materials.

Despite this, after assessment of the current state of this up-and-coming field, there are clear challenges that remain in their widespread adoption. Bias must continue to be carefully considered at every step of implementation. DoE is limited to linear regression, preventing it from establishing more complex nonlinear models, or classification models for categorical responses, so revealing the mechanistic insights of a reaction relies on researcher capability and accurate analysis of the results. In machine learning, both supervised and unsupervised models may be biased due to the way they were constructed or how they analyze and learn from the data. The remaining issues of overfitting and underfitting in machine-learning models demonstrate this. Additionally, the trade-off between time, expense, and accuracy must be carefully weighed for each specific investigation. For example, if the main goal is the realistic use of a certain material in a target application, time and expenses must be rationalized for that goal, which often come at the expense of the level of accuracy. The limitations in data collection when constructing training data sets are directly influenced by this, where the expense and cost of large data sets may outweigh the insights gained. This is exacerbated by the fact that data mining from the published literature is extremely challenging because of inconsistent experimental reporting, compounded by challenges in the automatic extraction of machine-readable data. These issues often lead to data-mining efforts being incomplete or requiring a large amount of manual cleaning.

Because of this, at present, the development of high-throughput, high-data volume experimentation and characterization methods are critically needed to solve limitations with machine learning for materials design, synthesis, and discovery. Initial efforts include combining robotic synthesis and self-driven laboratories guided by machine learning for autonomous materials discovery and optimization,^77,78^ but despite the growing popularity of robotic systems for chemical synthesis, they can be difficult to run and maintain due to a lack of standard operating systems and a capacity for direct access to the literature through natural language processing.^79^ In the future, implementing widespread guidelines for reporting scientific results, creating a universal system for data digitization, and making raw data freely available will advance our ability to data-mine and construct the large data sets needed for machine learning. Although the intuition of experienced chemists will always be required to appropriately identify problems and design the experiments that such autonomous systems will carry out,^80^ such advances will ultimately lead to a greater feasibility to model multivariate systems and solve problems that include mixed variable types and units of measurement. Additionally, computer vision will play a big role in improved characterization for electron microscopy analysis and could open the door for the implementation of machine learning across a wide range of analytical methods and problems in materials chemistry.^43,81−83^ Efforts to develop more user-friendly software to integrate common machine-learning models and toolboxes can make these techniques more accessible to chemists by reducing the time and effort spent on coding.
